# Genetic and Epigenetic Regulation of Cardiac Development: An Integrative View from Embryo to Human Pluripotent Stem Cell Models

**DOI:** 10.3390/cells15151316

**Published:** 2026-07-23

**Authors:** Swathy Babu

**Affiliations:** 1 Institute for Stem Cell Science and Regenerative Medicine (inStem), GKVK-Post, Rajiv Gandhi Nagar, Bengaluru 560065, Karnataka, India; babu0022@umn.edu; 2 Department of Neurology, University of Minnesota Twin Cities, Minneapolis, MN 55455, USA

**Keywords:** cardiogenesis, human pluripotent stem cell-derived cardiomyocytes, signaling pathways, cardiac transcription factors, epigenetic mechanisms, cardiovascular diseases

## Abstract

**Highlights:**

**What are the main findings?**

- Provides a comprehensive overview of the signaling pathways, transcription factors, and epigenetic mechanisms that regulate in vivo cardiac development and in vitro cardiomyocyte generation.
- Integrates insights from human embryonic heart development with in vitro cardiomyocyte differentiation strategies, emphasizing the molecular mechanisms underlying functional cardiomyocyte generation.

**What are the implications of the main findings?**

- Provides a framework for improving stem cell–based cardiomyocyte differentiation protocols by leveraging key genetic and epigenetic regulatory mechanisms.
- Highlights the translational potential of these developmental insights for disease modeling, drug discovery, and regenerative therapies targeting cardiovascular diseases.

**Abstract:**

Cardiogenesis is an exquisitely complex developmental process that unfolds through a series of tightly regulated and dynamic morphogenetic events. Proper heart development demands meticulous coordination of multiple signaling pathways, transcription factors and epigenetic mechanisms at every stage of cardiogenesis. Better understanding of the molecular regulation of cardiac development and the advances in the field of stem cell technology have paved way to the development of robust differentiation strategies to generate cardiomyocytes from pluripotent stem cells. This review provides a concise overview of key processes underlying human cardiac development, with a focus on their genetic and epigenetic regulation. It further discusses the generation of cardiomyocytes from human pluripotent stem cells, highlighting state-of-the-art differentiation protocols and detailing the genetic and epigenetic mechanisms that govern in vitro cardiomyocyte formation. By integrating insights from both in vivo human cardiac development and in vitro pluripotent stem cell-derived cardiomyocyte models, this article offers a comprehensive perspective on how embryonic cardiogenesis can be recapitulated in vitro, deepening our understanding of the molecular framework governing cardiomyocyte formation. Importantly, these insights hold significant translational potential for advancing disease modeling, drug discovery, and regenerative therapies for cardiovascular diseases.

## 1. Introduction

The development of the heart and circulatory system results from a series of highly complex processes involving combinatorial interactions among multiple signaling pathways, transcription factors, and their downstream target genes that co-ordinately regulate cardiac cell fate, differentiation and morphogenesis. In addition to these signaling networks and transcriptional regulators, epigenetic mechanisms including DNA modifications, histone modifications, chromatin remodeling and RNA-based regulation via non-coding RNAs such as microRNAs (miRNAs) and long non-coding RNAs (lncRNAs) play a critical role in cardiac differentiation. Studies using in vitro cell culture systems and animal models have provided a wealth of information on the molecular and epigenetic mechanisms underlying cardiac development, offering significant insights into the pathophysiology of cardiac diseases and potential therapeutic strategies [1,2,3,4,5].

The integration of knowledge from embryonic heart development and stem cell biology has enabled the development of strategies to generate cardiomyocytes from pluripotent stem cells in vitro. It is generally accepted that the differentiation of pluripotent stem cells into functional cardiomyocytes follows a developmental paradigm similar to that of embryonic cardiogenesis [6]. Over the past two decades, numerous efficient protocols have been established to derive cardiomyocytes from embryonic stem cells (ESCs) and induced pluripotent stem cells (iPSCs) [7,8]. These pluripotent stem cell-derived cardiomyocytes (PSC-CMs) have been extensively utilized for disease modeling of cardiomyopathies and channelopathies, as well as for regenerative therapies, drug discovery and cardiotoxicity screening. A deeper understanding of the molecular mechanisms underlying cardiomyocyte generation serves as a valuable foundation for developing more robust and reliable strategies to generate PSC-CMs for basic, clinical and translational research.

This review provides a concise overview of the stages of in vivo cardiac development alongside current strategies for generating cardiomyocytes from human PSCs in vitro. In particular, it emphasizes the genetic and epigenetic mechanisms that govern and coordinate these processes. By bridging developmental biology with stem cell-based models, this review article enhances our ability to translate fundamental insights into improved disease modeling, targeted drug discovery, and the development of regenerative therapies for cardiovascular diseases.

## 2. Human Cardiogenesis

### 2.1. Stages of Cardiogenesis

The heart is the first functional organ to form during development in all vertebrate embryos. In humans, the heart begins to beat and pump blood at around day 21 or 22 after fertilization, whereas in mice this occurs at approximately embryonic day 8 [6]. Cardiac development begins during gastrulation, when epiblast cells migrate through the primitive streak to generate mesodermal lineages, including the lateral plate mesoderm. Within the splanchnic layer of the lateral plate mesoderm, cardiogenic mesoderm is specified, and multipotent cardiac progenitor cells migrate to form the bilateral heart fields, which merge to generate the cardiac crescent (approximately day 15 in humans and E7.5 in mice) [9]. The cardiac crescent consists of first heart field cells laterally and second heart field cells medially, which proliferate and give rise to different cardiac structures [10].

During the third week of development in humans (approximately between day 19–21, corresponding to E8 in mouse), cardiogenic cords appear in the cardiogenic mesoderm and canalize to form two endocardial heart tubes, which further coalesce into a single primitive heart tube by lateral embryonic folding [11]. The primitive heart tube is made up of three layers: the endocardium, forming the inner endothelial lining; the myocardium, forming the mid muscular layer; and the visceral pericardium, forming the outer surface of the embryonic heart tube [12]. The heart tube subsequently elongates through the progressive addition of secondary heart field progenitor cells and develops into five distinct regions, from cranial to caudal: truncus arteriosus, bulbus cordis, primitive ventricle, primitive atrium, and the sinus venosus. The truncus arteriosus will give rise to the aorta and pulmonary trunk; the bulbus cordis and the primitive ventricle form the ventricles; the primitive atrium forms the atria; and the sinus venosus contributes to the sinus venarum, coronary sinus, sinoatrial node and oblique vein of the left atrium. By day 22 of human development (E8.5 in mouse), peristaltic contractions of the heart tube propel blood from the sinus venosus towards the truncus arteriosus, a circulatory pattern markedly different from that of the adult heart [13].

The primitive heart undergoes a cardiac looping process late in the fourth week to early in the fifth week of development (approximately days 23–28 in humans; E8.5–E10.5 in mouse), during which it curves to form a C-shape with its convexity directed towards the right side of the embryo and then an S-shape, forming a structure reminiscent of the adult heart [14,15,16]. Cardiac looping makes distinguishable the four anatomical segments, namely the atrium, atrioventricular canal (AVC), ventricle, and outflow tract (OFT), bringing them into close proximity for the remainder of cardiac development [15]. Immediately following looping, endocardial cushions develop in the atrioventricular canal and conotruncal region of the bulbus cordis; these cushions serve as primordia for the membranous septa and valves. Between days 29 and 37 (E11.0–E14.5 in mice), the heart segments undergo septation, partitioning the heart into four chambers (right atria, right ventricle, left atria, left ventricle) and dividing the OFT into aortic and pulmonary roots [14,17,18]. The formation of the atrioventricular (mitral and tricuspid) and semilunar valves (aortic and pulmonic) occurs between fifth and ninth weeks of development (E12–E17 in mice), facilitating the unidirectional blood flow through the heart [14]. In addition to mesoderm-derived cardiac progenitors, cardiac neural crest cells (cNCCs) and epicardial progenitors contribute significantly to cardiac development, with cNCCs involved in outflow tract remodeling, septation, and valve formation, and epicardial progenitors contributing to coronary vasculature, cardiac fibroblast, and vascular smooth muscle lineages [9,17].

**Figure 1:**
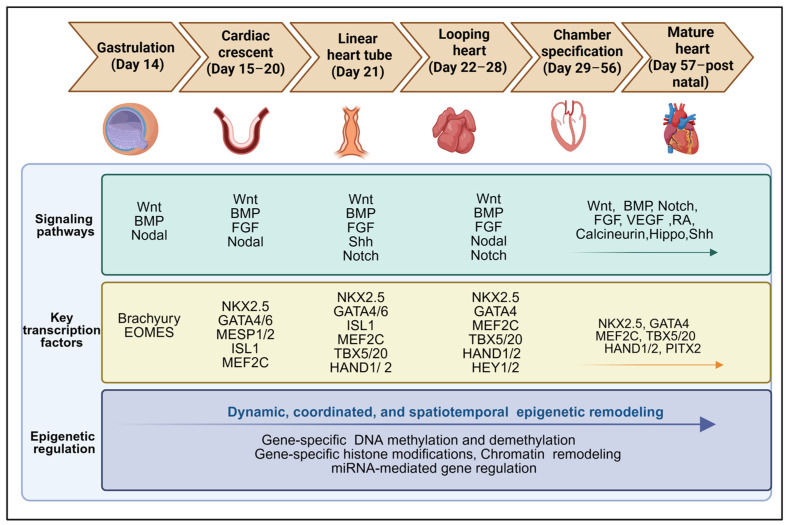
Key Stages and Regulatory Networks in Cardiac Development. Schematic representation of the sequential stages of cardiac development, beginning with gastrulation and progressing through cardiac crescent formation, linear heart tube formation, heart looping, chamber specification, and maturation of the functional heart. At each developmental stage, distinct but overlapping signaling pathways—including Wnt, BMP, Nodal, FGF, Shh, Notch, VEGF, RA, calcineurin, and Hippo signaling—coordinate cardiac lineage specification, morphogenesis, and chamber formation. These signaling networks regulate the temporal activation of key cardiac transcription factors, including Brachyury, EOMES, NKX2.5, GATA4/6, MESP1/2, ISL1, MEF2C, TBX5/20, HAND1/2, HEY1/2, and PITX2, which collectively drive cardiac progenitor specification, differentiation, and maturation. Throughout these developmental stages, epigenetic mechanisms, including DNA methylation, histone modifications, chromatin remodeling, and miRNA-mediated gene regulation, dynamically coordinate gene expression programs required for normal cardiogenesis and heart maturation.

Concurrent with the basic chamber formation, the coronary vasculature and cardiac conduction system develop. The components of the cardiac conduction system include the sinoatrial (SA) node, the atrioventricular (AV) node, bundle of His, bundle branches, and Purkinje fibers. The conduction system functions to generate electrical pulses and coordinate the contractions of cardiac chambers [19]. By approximately day 50 of human development, the heart reaches its final anatomical configuration, although structural refinement and functional maturation continue throughout gestation and after birth. The transition from fetal to neonatal circulation is accompanied by intricate physiological and anatomical changes in the cardiovascular system [20]. Cardiac embryological events have been reviewed in detail elsewhere [15,21,22,23]. Key stages of cardiac development are illustrated in Figure 1.

### 2.2. Signaling Pathways and Transcriptional Networks in Cardiogenesis

#### 2.2.1. Signaling Pathways

Early embryonic events of cardiogenesis are remarkably conserved from insects to vertebrates, allowing observations made in invertebrate and vertebrate model organisms to be extrapolated to humans. There has been an explosion of studies using embryo models and stem cell systems to understand the signaling and transcriptional networks that control cell lineage specification and heart morphogenesis [24,25,26]. Several morphogenic pathways including Wnt, bone morphogenic proteins (BMPs), fibroblast growth factors (FGFs), Notch, Nodal, Calcineurin/NFAT, Retinoic acid (RA), vascular endothelial growth factor (VEGF), Sonic hedgehog (Shh) and Hippo-YAP have been implicated at various stages of cardiogenesis. The major signaling pathways involved at various stages of cardiac development are summarized below and depicted in Figure 1.

Wnt signaling is a key regulator of vertebrate heart development. Earlier studies have reported apparently contradictory effects of Wnt signaling on cardiogenesis, depending on the model system used. In chick and xenopus embryos, Wnt signaling inhibited cardiac development line [27,28], whereas it promoted cardiogenesis in drosophila and P19CL6 mouse embryonal carcinoma cell line [29,30]. However, evidence from mouse embryonic stem cells suggests that canonical Wnt signaling exhibits a stage-specific biphasic role in cardiac development, promoting cardiac gene expression during early mesoderm formation and inhibiting later cardiac differentiation [31]. Early activation of canonical Wnt/β-catenin is essential for primitive streak formation, epithelial to mesenchymal transition and mesoderm induction [32]. Key Wnt ligands such as Wnt-3a and Wnt-8a regulate mesoderm induction via canonical signaling [31]. Inhibition of endogenous Wnt activity results in abrogation of functional development of mature mesodermal lineages [33]. In contrast, Wnt 5a and Wnt11 stimulates cardiomyocyte formation and differentiation by activating noncanonical pathways and simultaneously inhibiting the canonical Wnt/β-catenin pathway [34,35]. Wnt signaling is also known to crosstalk with multiple other pathways—including BMP, FGF, and Notch, during cardiac specification and differentiation [36].

Studies in vertebrate and invertebrate animal models have implicated a positive regulatory role for BMP signaling, acting at multiple stages to control the differentiation, proliferation, and morphogenesis of various cardiac tissues. Genetic studies in mice have shown that Bmp 4 is required for mesoderm formation and Bmp4-deficient mice failed to develop mesoderm [37]. Mice deficient for Bmp2 are embryonically lethal and showed cardiac defects, while Bmp10 knockout mice show reduced cardiomyocyte proliferation [38]. Noggin-mediated antagonism of BMP signaling has been shown to completely block the differentiation of precardiac mesoderm [39]. Evidence from genetically modified mouse models indicates that BMP5, BMP6, and BMP7 play critical roles in later stages of cardiac morphogenesis and chamber formation [40].

FGF signaling exhibits stage-dependent biphasic roles in cardiogenesis, promoting mesoderm formation and cardiac lineage commitment at early stages while inhibiting cardiac progenitor cell differentiation at later stages [41]. FGF signals, alone or in combination with BMP, regulate cardiac gene expression and early cardiogenesis [42]. Early activation of FGF and Nodal pathways has been reported to mediate cardiac specification independently of Wnt/β-Catenin signaling [43]. Multiple FGF ligands, including FGF1, FGF2, FGF3, FGF4, FGF7, FGF8, FGF9 and FGF10 have been reported to exert functional and structural effects during heart development [44].

Notch signaling in the endocardium is known to regulate cardiac specification, progenitor cell differentiation, and tissue patterning in the developing heart [45]. During the early stages of heart development, Notch signaling contributes to determining the fate of cardiac progenitor cells. In later stages of cardiac morphogenesis, the pathway regulates the formation of multiple cardiac structures, including the atrioventricular canal, endocardial cushions, and the cardiac conduction system, and also contributes to the development of the sinoatrial node [46].

In addition to the pathways discussed above, other signaling cascades—including Nodal, calcineurin/NFAT, RA, VEGF, Shh, and Hippo–YAP signaling—also contribute to the complex regulatory network that regulates cardiogenesis. Nodal signaling, mediated through its cofactor Cripto and the type I receptor Alk4, is critical for early cardiac specification in embryonic stem cells [47]. Activation of Nodal signaling engages the Smad2 pathway, and its inhibition by Nodal antagonists blocks cardiomyocyte induction, highlighting its essential role in early cardiogenesis. Calcineurin signaling and NFAT activation regulate the adult cardiac hypertrophic response, as well as valvuloseptal and vascular development [48]. Retinoic acid (RA) signaling plays an important role in regulating anteroposterior patterning, the formation of inflow and outflow tract progenitors, and the growth of the ventricular compact wall [49]. VEGF signaling plays a critical role in multiple aspects of cardiovascular development, including endothelial cell differentiation, migration and survival as well as heart formation and hematopoiesis [50]. Shh signaling is a crucial player in early heart development by regulating the proliferation, migration, and specification of cardiac progenitor cells, particularly in the second heart field [51]. Shh is also essential for proper outflow tract formation, atrial and ventricular septation, and overall cardiac morphogenesis. The Hippo–YAP pathway is yet another key regulator of heart development and cardiac homeostasis, controlling cardiomyocyte proliferation, heart size, and chamber formation. It also orchestrates multiple processes during cardiac regeneration, including cardiomyocyte proliferation and differentiation, stress responses, mechanical signaling and injury resistance [52].

#### 2.2.2. Transcriptional Networks

Downstream of signaling pathways, a diverse array of transcription factors regulates cardiac cell fate and differentiation by orchestrating spatiotemporal gene expression programs. A comprehensive overview of key transcription factors with established roles in cardiac development is outlined below and presented in Figure 1.

The formation of the primitive streak is defined by the expression of Brachyury and EOMES, representing the earliest known lineage-specifying transcription factors. Brachyury and EOMES promote the formation of early cardiac mesoderm by directly activating the basic-helix-loop-helix transcription factor genes *MESP1* and *MESP2* [53,54]. MESP1 acts as a master regulator of cardiovascular progenitor specification and activates numerous genes within the core cardiac transcriptional machinery. Mutations in *TBXT* (encoding Brachyury), *EOMES*, and *MESP1* have been shown to impair the development of the mesoderm and mesoderm-derived structures [55,56,57].

The mesodermal cells expressing cardiac-specific transcription factors undergo progressive specification into the cardiac lineage, giving rise to cardiac progenitor cells that subsequently differentiate into multiple cell types within the heart. A subset of the MESP1^+^ mesodermal cells express the NK-2 homeobox transcription factor NKX2.5 and LIM homeobox transcription factor ISL1, which are early markers for cardiac cell specification and proliferation. Lineage tracing studies in mouse models indicate that Nkx2.5 and Isl1 expressing precursors contribute extensively to cardiomyocyte, smooth muscle and endothelial lineages [58]. Another population, marked by Flk1/KDR and PDGFRα, represents an early mesodermal subset enriched for cardiac mesoderm cells [59]. Upon activation by MESP1, the expression of key cardiac transcription factors—including NKX2.5, GATA4, GATA6, HAND2, MEF2C, TBX20, and Myocardin—is promoted. NKX2.5 appears to act cooperatively with transcription factors such as GATA4, MEF2C, and TBX5 to drive cardiomyocyte differentiation. In particular, NKX2.5 and GATA4 physically interact and synergistically activate several cardiac genes, including α-cardiac actin and the cardiac-restricted ankyrin repeat protein [60]. Functional and physical interactions among various core cardiac transcription factors tightly regulate cardiac gene expression [61,62,63]. Mutations in these transcription factor genes result in defects in cardiomyocyte differentiation and proliferation [64,65].

An expanding network of transcription factors—including ISL1, MEF2C, HAND 2, FOXH1, FOXC1/C2, TBX1 and HES1—has been identified as essential for the development of the second heart field and its derivatives. At least six members of the T-box gene family, namely TBX1-3, TBX5, TBX18 and TBX20, are expressed in distinct compartments of the developing heart [66,67]. Additionally, numerous other transcription factors and regulatory factors, including Myocardin, MEF2A/C, MSX2, SRF, HEY1/2, PITX2, PAX3, PBX1-3, TWIST1, SOX4, SOX11 and IRX4, play specific roles in heart development. Mutations of several cardiac-specific transcription factors in heart development cause a myriad of congenital heart diseases [68,69,70,71].

### 2.3. Epigenetic Regulation in Cardiac Development

Epigenetics, defined as heritable modifications of gene expression independent of DNA sequence changes, has emerged as a pivotal regulator of cardiovascular development and function. This regulation involves DNA methylation and histone modifications, dynamic changes in chromatin structure through chromatin remodeling as well as the regulatory functions of non-coding RNAs, particularly miRNAs. Dynamic epigenetic changes occur throughout human cardiac development, and the key epigenetic mechanisms are discussed below (Figure 1).

#### 2.3.1. DNA Methylation

Epigenetic regulation through DNA methylation plays a central role in shaping cardiac development and function. During the early stages of cardiac specification and cardiogenesis, dynamic DNA methylation remodeling occurs at regulatory regions of key developmental genes, contributing to the transition of pluripotent progenitor cells toward cardiac lineages by modulating the accessibility and transcriptional activity of cardiac fate determinants [72]. Studies in the mouse embryonic heart have shown that global DNA methylation level remains stable between E11.5 and E14.5; however, differential methylation occurs in a subset of genes, correlating with changes in the expression of key cardiac genes, including *Has2*, a gene essential for epicardial cell differentiation, heart valve development, and septation [73]. In addition, Dnmt3b is shown to regulate Has2 expression, potentially through enhancer methylation. Beyond early specification, DNA methylation patterns continue to be remodeled during cardiomyocyte differentiation and postnatal maturation [72,74]. Demethylated regions in neonatal and adult cardiomyocytes are predominantly localized to cell type-specific enhancer elements and within the gene bodies of cardiomyocyte-specific genes. DNA methylation plays a key role in regulating the expression of troponin I (TnI) isoforms, particularly slow skeletal TnI, which is predominantly expressed in the fetal heart [75]. Loss of CIBZ, a methyl-CpG binding protein, promotes mesodermal and cardiac differentiation in mouse ESCs by inducing the expression of Brachyury and Mesp1 expression [76].

Several studies have investigated the roles of various epigenetic enzymes in regulating cardiac development and function. Knockdown of *Dnmt3a* in embryonic mouse hearts (E13.5) disrupted sarcomere assembly, and reduced contractility, beating frequency, field action potential and intensity of calcium signaling [77]. *Dnmt3a* disruption also affected multiple pathways related to cardiac function, including those regulating heart rate, contraction, cardiogenesis, cardiac muscle function, morphology, and sarcomere organization. Knockdown of DNMT1 resulted in a reduction in global DNA methylation, whereas knockdown of DNMT3a or DNMT3b did not significantly affect global methylation levels. However, depletion of DNMT3a or DNMT3b induced gene-specific methylation changes in multiple cardiac genes, which correlated with altered gene expression. Cardiac-specific deletion of *Dnmt3b* in adult mouse hearts correlated with alternative splicing of the sarcomeric gene Myh7 [78].

Disruption of *Dnmt1* in embryonic mouse cardiomyocytes reduced gene-specific DNA methylation in several cardiac genes, including *Myh6*, *Tnnc1*, *Tnni3*, *Tnnt2*, *Nppa* and *Nppb* [79]. A study in myocardial tissue-specific *Dnmt1* knockout rats demonstrated that *Dnmt1* deficiency is associated with injury resistance and protection in the myocardium under pathological conditions [80]. Dynamic modulation of DNA hydroxymethylation is linked to transcriptional networks governing heart development, with the enzyme ten-eleven translocation 2 (TET2) regulating key cardiac genes, such as *MYH7*, through 5-hmC deposition on gene bodies and enhancers [81]. Studies in zebrafish larvae demonstrated that Tet2/3-dependent demethylation is required for the expression of *inhbaa* and *sox9b*, genes involved in atrioventricular canal extracellular matrix organization, thereby controlling epicardial progenitor cell migration onto the developing heart tube [82].

#### 2.3.2. Histone Modifications and Chromatin Remodeling

Several lines of evidence indicate that histone acetylation and deacetylation play a critical role in cardiac development. The histone acetyltransferase (HAT) p300 regulates the expression of numerous cardiac-specific transcription factors, including GATA4, GATA5, MEF2C and NKX2.5 [83,84]. Deficiency or aberrant expression of p300 is associated with defective heart development, leading to embryonic lethality during early gestation [85]. MOZ, another HAT, acts through H3K9 acetylation and regulates the transcriptional activity of the *Tbx1*, *Tbx2*, and *Tbx5* loci in vivo [86]. Cardiac-specific deletion of both *Hdac1* and *Hdac2* resulted in neonatal lethality, accompanied by upregulation of genes encoding calcium channel and skeletal muscle contractile protein genes, suggesting redundant roles of HDAC1 and HDAC2 in cardiac growth and development [87]. Similarly, mouse embryos lacking *Hdac3* in cardiac progenitor cells exhibit precocious cardiomyocyte differentiation, severe cardiac developmental defects, upregulation of Tbx5 target genes and embryonic lethality [88]. HDAC5 and HDAC9 also display functional redundancy; their combined deletion leads to embryonic or early postnatal lethality due to cardiac abnormalities including ventricular septal defects and thin-walled myocardium [89]. In contrast, myocyte-specific overexpression of *Hdac3* in mice promotes cardiomyocyte proliferation through inhibition of cyclin-dependent kinase inhibitors [90]. Furthermore, knockout of the class III HDAC-encoding gene *Sirt1* results in perinatal or postnatal lethality, primarily due to septal defects [91].

Histone methylation is another important post-translational modification that influences cardiac development. SMYD1, a histone methyltransferase (HMT), is essential for the proper expression of the cardiac gene program in both early and late stages of heart development. SMYD1-mediated histone methylation modulates the expression of key cardiac transcription factors, including Hand2 and Irx4 [92], and also plays a role in myofibril organization and muscle contraction in zebrafish embryos [93]. SETD2, an H3K36me3 methyltransferase, is essential for coronary vessel formation and the overall heart development, as evidenced by mice that lack Setd2, which display defects in coronary vasculature and ventricular non-compaction [94]. Whsc1, another HMT, negatively regulates NKX2.5 and its downstream target genes, possibly through histone H3 trimethylation at lysine 36 (H3K36me3) [95]. Deletion of Ptip, a key component of the H3K4me complex, in adult cardiomyocytes results in defects in the cardiac conduction system and increased susceptibility to Ca^2+^-mediated ventricular arrhythmias [96]. The Polycomb histone methyltransferase EZH2 mediates repression of Six1 in differentiating cardiac progenitors, which is essential for maintaining stable postnatal cardiac gene expression and homeostasis [97]. Histone lysine methyltransferase G9a forms a repressive complex with MEF2C and HDAC5 to regulate sarcomere organization [98]. Cardiac-specific knockout of Dot1L, leads to increased mortality, accompanied by chamber dilation, elevated cardiomyocyte cell death, systolic dysfunction, and conduction abnormalities, thereby establishing the importance of Dot1L-mediated H3K79 methylation in cardiomyocyte function [99]. Mll2 regulates cardiac lineage differentiation of mouse ESCs by promoting H3K4me3 deposition at core cardiac-specific genes [100].

Several histone demethylases have been implicated in cardiac development. UTX, a H3K27 demethylase, functions as a coactivator of core cardiac transcription factors, including SRF, TBX5, NKX2.5 and GATA4, and acts as a critical switch to activate the cardiac developmental program [101]. UTX-deficient ESCs fail to develop heart-like rhythmic contractions during cardiac differentiation, and UTX-deficient mice exhibit severe defects in heart development and embryonic lethality. Mice carrying a hypomorphic variant of *LSD1* display highly penetrant defects in the formation of the ventricular septum [102]. Members of the family of JmjC domain proteins are also linked to cardiac development. H3K27 demethylases Jmjd3, is required for mesoderm differentiation and cardiovascular lineage commitment in mouse embryonic stem cells [103]. Ablation of Jmjd6 resulted in ventricular septal defect and double outlet right ventricle in mice [104]. JARID2, a key regulator of histone trimethylation, is expressed in both the developing and adult heart, and Jarid2 knockout mice exhibit cardiac malformations [105]. Early deletion of Jarid2 in cardiac progenitors prior to cardiomyocyte differentiation results in morphogenetic defects manifested in later stages of development [106].

Investigations into the roles of SWI/SNF chromatin remodeling complexes, including BAF and PBAF, indicate that ATP-dependent chromatin remodeling is critical for cardiac differentiation. The dosage of Brg 1, an ATPase subunit of the BAF complex, is critical for cardiogenesis in both mice and zebrafish [107]. Disruption of the balance between Brg1 and cardiac transcription factors, including Tbx5, Tbx20, and Nkx2-5, leads to severe cardiac anomalies [108]. Brg 1 interacts with HDAC and PARP to repress α-MHC and activate β-MHC expression [109]. BAF60C, a subunit of the BAF complexes, is required for normal heart morphogenesis and establishment of left–right asymmetry in mouse heart [110]. BAF180, a key component of the PBAF chromatin-remodeling complex, is essential for mammalian cardiac chamber maturation, with its deficiency leading to severe ventricular hypoplasia and embryonic lethality between E12.5 and E15.5 [111]. The essential roles of other subunits of the BAF complex subunits, including BAF47, BAF155, and BAF250, have also been documented in cardiac development [112,113].

#### 2.3.3. MicroRNA Expression

MicroRNAs are critical regulators of cardiac growth and function. miR-1 is the most abundant miRNA in CMs and adult heart tissue and it regulates a broad spectrum of genes involved in cardiac development, including *HAND2*, *HDAC4*, *IRX5*, *GJA1*, and *KCNJ2* [114,115]. Moreover, it plays an essential role in sarcomere formation and suppresses smooth muscle gene expression in the heart [116]. Double knockout mice for both mature forms of miR-133a exhibit ectopic smooth muscle gene expression, aberrant cardiomyocyte proliferation, ventricular septal defects, and cardiomyopathy, highlighting the essential and redundant roles of miR-133a in cardiac development and function [117]. Additionally, miR-133 and miR-30 jointly regulate myocardial structural remodeling by targeting *CTGF*, a profibrotic factor, thereby modulating extracellular matrix dynamics [118]. miR-138 is required for establishing appropriate chamber-specific gene expression patterns during zebrafish heart development [119].

miR-145 and miR-143 cooperatively regulate multiple transcription factors, including KLF4, myocardin, and ELK-1, thereby promoting differentiation and repressing proliferation of vascular smooth muscle cells [120]. The miR-17/92 cluster, which comprises six members—miR-17, miR-18a, miR-19a, miR-19b-1, miR-20a, and miR-92a-1—is essential for cardiac development, as global knockout of this cluster results in cardiac septal defects and neonatal lethality [121]. The miR-322/503 cluster is enriched in the early cardiac progenitor cells, is specifically expressed in the developing heart tube, and drives precocious cardiomyocyte specification [122]. Reduced expression of miR-940 disrupts the proliferation and migration of secondary heart field progenitor cells by targeting JARID2 [123]. miR-21 is recognized for its involvement in vascular smooth muscle cell proliferation, cardiac cell growth and apoptosis, and cardiac fibroblast functions [124], while miR-23 is required to restrict endocardial cushion formation by inhibiting Has2 expression and extracellular hyaluronic acid production [125].

Recently, muscle-specific miRNAs, known as MyomiRs, have emerged as critical regulators of cardiovascular development. The canonical MyomiRs-miR-1, miR-133a/b, and miR-206, along with miR-208a/b and miR-499, are abundantly expressed in the myocardium and regulate certain aspects of physiological and pathological processes in myocardiocytes including cardiovascular development, myocardial remodeling, and cardiovascular disease progression [126].

## 3. Differentiation of Human Pluripotent Stem Cells into Cardiomyocytes: Methodologies and Underlying Molecular Mechanisms

Over the past two decades, numerous strategies have been developed to derive cardiomyocytes from pluripotent stem cells, aiming to faithfully recapitulate early embryonic developmental processes in vitro through the modulation of cardiac-specific signaling pathways. In vitro cardiac differentiation proceeds through a series of sequential events, beginning with mesoderm induction, followed by patterning into cardiac mesoderm, differentiation into cardiac progenitors, and ultimately maturation into functional cardiomyocytes. Conceptually, the same signaling pathways that regulate embryonic heart development—such as Wnt, FGF, BMP, and Activin/Nodal—are utilized to direct PSC differentiation into cardiomyocytes in vitro [6]. Stem cell-derived cardiomyocytes exhibit key functional and molecular characteristics of native cardiomyocytes, including spontaneous contractile activity and the expression of cardiac-specific proteins, ion channels, and signaling molecules.

### 3.1. Approaches for Cardiomyocyte Differentiation from PSCs

**Figure 2:**
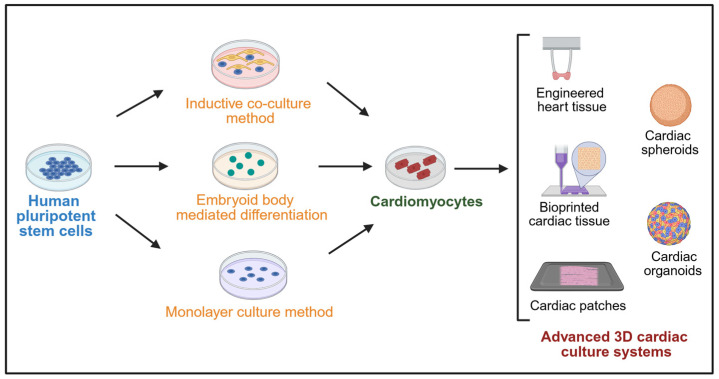
Overview of differentiation strategies and advanced 3D cardiac culture systems for generating cardiomyocytes from hPSCs. hPSCs can be differentiated into cardiomyocytes using inductive co-culture (pioneering approach), EB-mediated differentiation (early approach), or monolayer culture methods (current standard approach). The resulting cardiomyocytes can subsequently be incorporated into advanced 3D cardiac culture systems, including engineered heart tissues, bioprinted cardiac tissues, cardiac patches, cardiac spheroids, and cardiac organoids.

Three major approaches are commonly employed to derive cardiomyocytes from human PSCs: (i) Inductive co-culture, (ii) Embryoid body-mediated differentiation, and (iii) Monolayer culture systems (as illustrated in Figure 2).

The inductive co-culture approach, initially established using mouse visceral endoderm-like (END-2) cells, promotes cardiogenesis through paracrine signaling-mediated induction of cardiac fate. Although this method was among the earliest approaches used to derive cardiomyocytes from hESCs and mechanically passaged hiPSCs, its application has declined due to limited differentiation efficiency, reliance on feeder-like cells, and challenges in standardization and scalability [127,128,129,130,131].

The embryoid body (EB)-mediated approach involves the differentiation of stem cells through the formation of multicellular aggregates known as embryoid bodies, which are an amalgam of all three embryonic germ layers. In conventional EB protocols, spontaneous differentiation in suspension cultures or hanging-drop systems generates EBs that subsequently produce spontaneously contracting cardiomyocytes upon transfer to extracellular matrix-coated substrates [132,133,134,135,136,137,138]. Subsequently, several strategies have been developed to enhance the efficiency of EB differentiation, including controlled aggregation in V-bottom plates, engineered microwells, and microfabrication approaches, as well as modulation of biophysical cues, such as electrical stimulation and hypoxia [139,140,141,142,143,144]. Recent advances incorporating stirred suspension bioreactors, three-dimensional culture systems, and engineered aggregation platforms have enabled more uniform EB generation and scalable cardiomyocyte production [145]. In addition, temporal regulation of developmental signaling pathways using factors such as Activin A, BMP4, FGF2, VEGF, DKK-1, CHIR99021, and Wnt inhibitors has enhanced cardiomyocyte yield and maturation [146,147,148]. Transcriptomic studies suggest that three-dimensional EB-based differentiation promotes more developmentally relevant maturation trajectories compared with conventional two-dimensional approaches [144]. Despite these improvements, challenges including batch variability, process complexity, and scalability remain, driving the integration of EB platforms with advanced engineering approaches and precise biochemical modulation.

In parallel with EB-based approaches, monolayer culture platforms have emerged as a widely adopted directed differentiation strategy for cardiomyocyte generation, employing defined culture conditions and temporally controlled application of developmental cues to guide PSCs toward the cardiac lineage [149]. These protocols have evolved from growth factor-based approaches using defined, serum-free media to chemically defined small molecule-based strategies that enable more efficient and reproducible cardiomyocyte production from PSCs [150,151,152,153,154]. For example, modulation of developmental signaling pathways using combinations of growth factors, including BMP4, Activin A, FGF2, and VEGF, or small molecules targeting Wnt signaling, has significantly improved cardiac differentiation efficiency. Several optimized protocols derived from this principle have further improved cardiomyocyte yield, consistency, and scalability in hPSC differentiation systems [148,155,156].

Although hPSC-CMs closely resemble their in vivo counterparts, they generally exhibit an immature phenotype characterized by fetal-like structural, electrophysiological, metabolic, and contractile properties. Consequently, considerable efforts have been directed toward enhancing hPSC-CM maturation to increase their physiological relevance and translational applicability. These maturation strategies include prolonged culture duration, genetic manipulation, and the supplementation of miRNAs (e.g., miR-1) and biochemical factors such as triiodothyronine (T3), glucocorticoids (e.g., dexamethasone), phenylephrine, and insulin-like growth factor I [157]. Additional strategies include co-culture with non-cardiomyocytes (e.g., cardiac fibroblasts and endothelial cells), modulation of substrate topography and viscoelasticity, three-dimensional cardiac tissue engineering, and electrical and/or mechanical stimulation [158,159,160]. More recent studies have explored the integration of multiple maturation strategies to further enhance the functional and structural maturity of iPSC-CM. Lyra-Leite et al. provided a comprehensive review of protocols related to hPSC culture, cardiomyocyte differentiation, subtype specification, cardiomyocyte maturation, and somatic cell direct reprogramming, detailing the methodologies and critical considerations for generating functional cardiomyocytes from hPSCs [161].

To better recapitulate the native cardiac microenvironment and improve hiPSC-CM maturation, recent advances have shifted from traditional two-dimensional cultures to sophisticated three-dimensional cardiac constructs. These constructs encompass a range of platforms, including engineered heart tissues, cardiac spheroids, bioprinted cardiac tissues, cardiac patches, and cardiac organoids, which differ in their degree of cellular organization and complexity (Figure 2) [162,163,164,165,166,167,168,169,170,171,172]. By providing a physiologically relevant microenvironment that promotes cell–cell and cell–matrix interactions, three-dimensional cardiac constructs enhance the structural, electrophysiological, contractile, and metabolic maturation of hiPSC-CM compared with conventional two-dimensional cultures. Consequently, these advanced platforms have emerged as valuable tools for studying cardiac development, disease modeling, drug screening, and regenerative medicine applications [173,174,175,176,177,178,179,180,181,182,183,184].

**Figure 3:**
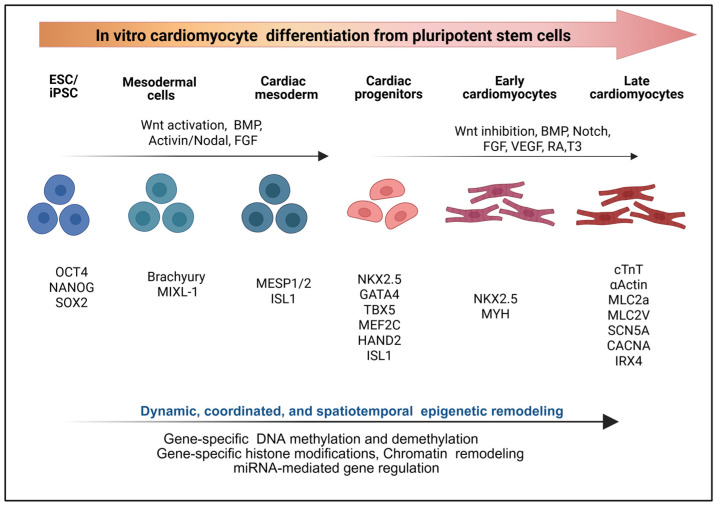
Overview of Cardiomyocyte Differentiation from hPSCs: Stages, Molecular Pathways, Key Transcription Factors, and Epigenetic Regulation. Schematic representation of the sequential progression from hPSC pluripotency to mature cardiomyocytes, highlighting distinct developmental stages including pluripotency, mesoderm induction, cardiac mesoderm specification, cardiac progenitor formation, early cardiomyocyte differentiation, and late cardiomyocyte maturation. Stage-specific modulation of developmental signaling pathways—including Wnt, BMP, Activin/Nodal, FGF, Notch, VEGF, RA, and T3—coordinates lineage commitment, cardiac specification, and structural and functional maturation. Key transcription factors associated with each stage include OCT4, NANOG, and SOX2 in pluripotent cells; Brachyury and MIXL1 during mesoderm induction; MESP1/2 and ISL1 during cardiac mesoderm formation; NKX2.5, GATA4, TBX5, MEF2C, HAND2, and ISL1 in cardiac progenitors; NKX2.5 and MYH during early cardiomyocyte differentiation; and mature cardiomyocyte markers, including cTnT, α-actinin, MLC2a, MLC2v, SCN5A, CACNA1C, and IRX4, during terminal maturation. Throughout this process, epigenetic mechanisms—including DNA methylation, histone modifications, chromatin remodeling, and miRNA-mediated regulation—coordinate dynamic gene expression programs that govern cardiac lineage commitment, differentiation, and functional maturation.

A schematic representation of the sequential stages of cardiomyocyte differentiation from hPSCs is presented in Figure 3.

### 3.2. Molecular Mechanisms Underlying Cardiac Differentiation from hPSCs

Regulation of multiple developmental signaling pathways, including Activin/Nodal, Wnt, BMP, FGF, and VEGF, has been extensively investigated and incorporated into differentiation methodologies to generate cardiomyocytes from hPSCs (Figure 3). Although considerable progress has been made in elucidating the molecular mechanisms underlying in vitro hPSC cardiac differentiation, the signaling networks and stage-specific markers governing these processes remain less comprehensively defined compared with those established during in vivo cardiogenesis, reflecting the relatively recent and rapidly evolving nature of hPSC-based differentiation systems. Nevertheless, numerous studies have identified key signaling pathways and temporal cues that regulate cardiac lineage specification and cardiomyocyte differentiation. For example, combined treatment with Activin A and BMP4 activates Activin/Nodal and BMP signaling pathways, respectively, to induce mesoderm formation in hPSC lines [152,185]. Treatment with CHIR99021 activates canonical Wnt/β-catenin signaling and promotes mesodermal differentiation [186,187]. As mentioned earlier, Wnt signaling exhibits a biphasic effect on cardiomyocyte differentiation: its activation promotes mesoderm formation, whereas its inhibition at later stages facilitates cardiac lineage commitment from the mesoderm. Treatment with FGF2, DKK1, and VEGF promotes the progression of mesodermal progenitors into cardiac mesoderm by modulating complementary signaling networks that regulate progenitor proliferation and lineage specification [188]. Various Wnt inhibitors including IWP2, IWP4, etc., are known to effectively lead to cardiac lineage commitment [186]. FGF10 promotes the proliferation and expansion of cardiac progenitors and is proposed to act through BMP, TGF-β, Wnt/β-catenin, Notch, and VEGF signaling pathways to induce cardiomyocyte differentiation [189].

Multiple signaling pathway-based strategies have been employed to direct the differentiation of iPSC-derived cardiac progenitors into specific cardiomyocyte subtypes. For instance, inhibition of neuregulin1β/ErbB signaling has been shown to increase the proportion of nodal pacemaker cells, whereas retinoid signaling directs atrial versus ventricular fate specification [190]. Additionally, inhibition of Nodal signaling at the cardiac mesoderm stage has been reported to promote the differentiation of hiPSCs into pacemaker-like cardiomyocytes. The cooperative action of BMP and retinoic acid, along with activation of canonical Wnt signaling favors pacemaker lineage [191]. Similarly, the combined activity of retinoic acid and Wnt signaling pathways regulates the differentiation of hiPSCs into sinoatrial node-like cells [192]. Furthermore, thyroid hormone signaling, primarily mediated by T3, plays a critical role in cardiomyocyte subtype specification and maturation of iPSC-CMs [193].

Revolutionary advances in single-cell multi-omics technologies, including single-cell RNA sequencing (scRNA-seq) and spatial transcriptomics, have significantly enhanced the understanding of molecular mechanisms governing cardiac differentiation from hiPSCs [194,195]. Single-cell transcriptomic studies have delineated dynamic differentiation trajectories of iPSC-derived cardiomyocytes and uncovered key regulatory networks driving lineage commitment. For example, scRNA-seq analyses have identified candidate genes such as *CREG* and *NR2F2* that play critical roles in cardiomyocyte specification and maturation [196]. Furthermore, integrative multi-omic profiling combined with machine learning approaches enables the prediction of early hPSC to CM differentiation efficiency and the identification of key cell fate determinants [197]. Such integrated analyses of progenitor cell states reveal molecular quality attributes that are predictive of downstream differentiation outcomes, thereby facilitating refinement of differentiation protocols and improving process robustness for efficient generation of functional cardiomyocytes [198,199]. Although the application of single-cell multi-omics to hPSC-derived cardiomyocyte differentiation is still an emerging field, rapid technological advances and the expanding body of literature are expected to uncover additional molecular, and spatial regulatory mechanisms governing cardiac lineage specification and maturation.

### 3.3. Epigenetic Mechanisms Regulating Cardiac Differentiation of hPSCs

Epigenetic mechanisms including DNA methylation, histone modifications and miRNA expression play a crucial role in the differentiation of hPSCs into cardiomyocytes (Figure 3). They are critical for maintaining pluripotency in hPSCs, as they establish an open chromatin state and demethylated promoters at key pluripotency genes like *OCT4*, *SOX2*, and *NANOG*, enabling their active expression and self-renewal while repressing differentiation-associated loci [200]. Differentiation of hPSCs to cardiomyocytes in vitro is accompanied by many notable epigenetic changes. Genome-wide DNA methylation analyses comparing hESCs and hESC-derived cardiomyocytes have revealed higher global DNA methylation levels in hESC-derived cardiomyocytes [201]. Notably, a subset of demethylated genes is upregulated during cardiomyocyte differentiation and is enriched for cardiac structural functions, indicating tight epigenetic control of structural gene expression. In contrast, cardiac-specific transcription factor promoters remain hypomethylated in both hESCs and hESC-derived cardiomyocytes, suggesting distinct epigenetic regulatory mechanisms governing transcription factors and structural genes.

A recent study showed that during differentiation of hPSCs into cardiomyocytes, exon-specific DNA methylation patterns are dynamically established in close association with transcriptional activity and lineage commitment [202]. These differentiation-associated methylation signatures are preferentially enriched within genes encoding developmental transcription factors, underscoring their regulatory significance. Importantly, the authors show that exon methylation persists even after gene silencing, suggesting that such marks serve as stable epigenetic “memories” of prior gene expression and cellular differentiation history.

A study profiling three histone modifications, H3K4me3, H3K27me3, and H3K36me3 across five stages of hESC-derived cardiovascular differentiation, from pluripotent cells to definitive cardiomyocytes, revealed that differentiation is accompanied by programmed temporal alterations in chromatin architecture [203]. These changes selectively mark key cardiac regulatory genes, including transcription factors and soluble signaling molecules, from lineage-specific genes encoding proteins that regulate cardiac function and homeostasis. Notably, many key transcription factors such as NKX2.5, GATA6, GATA4, HAND2 and TBX5, progressively lose the repressive mark H3K27me3, while gaining activating marks, H3K4me3 and H3K36me3, accompanied by stage-specific increases in transcription during differentiation. In contrast, structural genes such as MYH6 exhibit a pronounced gain of H3K4me3 enrichment and increased RNA expression at later stages, without early H3K27me3 repression. Furthermore, this study demonstrated that many of the members of key cardiac signaling pathways including Wnt, Notch, Shh, FGF, PDGF, and VEGF show stage-specific patterns of activation (H3K4me3, H3K36me3, RNA expression) and repression (H3K27me3), reflecting tight epigenetic control over the pathways that govern cell fate.

Emerging studies have demonstrated that dynamic miRNA expression patterns contribute to cardiomyocyte lineage specification, maturation, and the acquisition of adult-like functional properties during hPSC-to-cardiomyocyte differentiation. Members of the let-7 miRNA family have been identified as critical regulators of cardiomyocyte maturation, promoting adult-like metabolic remodeling and functional maturation in stem cell-derived cardiomyocytes [204]. Furthermore, comprehensive transcriptomic and miRNA profiling studies of differentiating hiPSC-derived cardiomyocytes have revealed temporally regulated miRNA signatures associated with cardiac lineage commitment, electrophysiological maturation, and metabolic adaptation [205,206]. Scalise et al. demonstrated that differentiation of adult cardiac stem/progenitor cells (CSCs) into contracting iCMs faithfully recapitulated the known cardiomyo-miR-dependent developmental cardiomyocyte differentiation trajectories, yielding a miRNome closely resembling that of adult cardiomyocytes [207]. Notably, several cardiomyocyte-enriched miRNAs (cardiomyomiRs), including miR-1a-3p, miR-133a-1, miR-133a-2, miR-204, miR-335, miR-486, miR-490, and miR-499, were upregulated in CSC-derived iCMs, with miR-1 and miR-499 further promoting myogenic commitment in vitro. Collectively, these findings highlight the role of temporally regulated miRNA networks in guiding cardiomyocyte differentiation and maturation. Several miRNAs that regulate each stage of in vitro iPSC-to-cardiomyocyte differentiation, as well as various cardiac transcription factors, have been reviewed elsewhere [208,209,210].

## 4. Comparative Overview of In Vivo Cardiogenesis and hPSC-CM Differentiation

Although cardiac development in the embryo occurs within a highly complex three-dimensional microenvironment, current hPSC differentiation protocols largely recapitulate the sequential developmental events that govern cardiogenesis. Key signaling pathways, including Wnt, BMP, Activin/Nodal, FGF, and VEGF, are temporally modulated in vitro to mimic their stage-specific functions during embryonic development, driving the progression from pluripotency through mesoderm induction, cardiac progenitor specification, and cardiomyocyte differentiation. Similarly, the activation of core cardiac transcription factors such as MESP1, NKX2.5, GATA4, TBX5, and MEF2C, together with dynamic epigenetic remodeling, reflects developmental programs observed during heart formation in vivo. This developmental parallelism provides a mechanistic framework for understanding how principles of embryonic cardiogenesis can be leveraged to generate cardiomyocytes in vitro and highlights the broad utility of hPSC-based models in basic, translational, and regenerative cardiovascular research.

**Figure 4:**
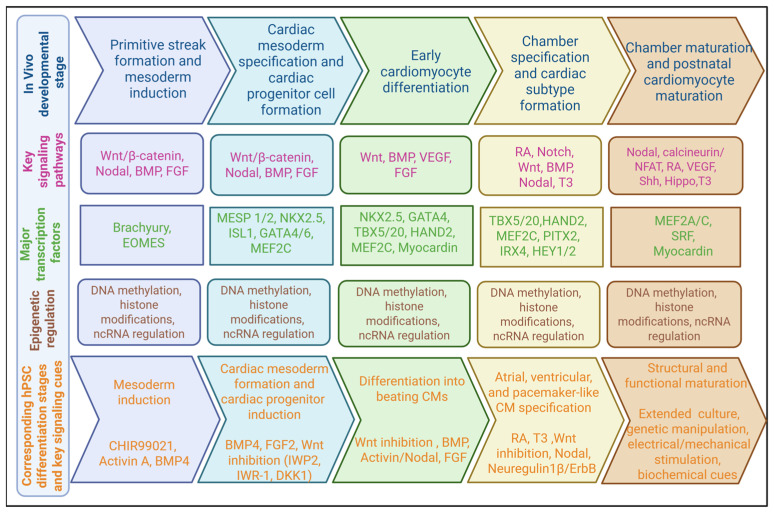
Comparative Overview of In Vivo Cardiogenesis and In Vitro hPSC-CM Differentiation. The schematic highlights shared developmental stages, signaling pathways, transcriptional regulators, and epigenetic mechanisms involved in embryonic heart development and hPSC-based cardiomyocyte generation, demonstrating the recapitulation of cardiac developmental programs in vitro.

Figure 4 provides a comparative overview of in vivo cardiogenesis and in vitro hPSC-CM differentiation. It highlights the conserved signaling pathways, transcription factors, and epigenetic mechanisms that regulate both embryonic cardiac development and hPSC-CM differentiation, demonstrating how key developmental processes are recapitulated in vitro to generate functional cardiomyocytes.

## 5. Conclusions

Cardiac embryogenesis is a multistage, highly dynamic developmental process governed by tightly coordinated cellular and molecular events. From early mesoderm commitment to the functional maturation of the human heart, precisely regulated signaling pathways, transcriptional networks, and epigenetic mechanisms act in concert to orchestrate cardiogenesis. Gaining a deeper insight into the molecular mechanisms governing heart development offers substantial benefits for both basic science and clinical applications. It enriches the broader field of developmental biology by shedding light on fundamental processes such as cell fate specification, tissue patterning, and organogenesis. Moreover, it enhances our understanding of the mechanisms underlying cardiac diseases and their pathology. Importantly, this knowledge facilitates earlier detection, improved risk stratification, and the development of targeted therapeutic strategies for both congenital heart diseases and acquired cardiovascular conditions.

Deciphering the molecular mechanisms underlying cardiac development significantly enhances the in vitro generation of cardiomyocytes from pluripotent stem cells by facilitating the recapitulation of key developmental signaling pathways and transcriptional networks. This knowledge allows for the optimization of differentiation protocols to improve efficiency, yield, and lineage specificity of cardiomyocyte subtypes. Furthermore, insights into epigenetic regulation help refine strategies to produce more stable and functionally relevant cardiomyocytes. Collectively, these advances strengthen the reliability and translational potential of in vitro cardiac models for disease modeling, drug discovery, and regenerative medicine applications.

Despite substantial advances in hPSC-CM platforms, several fundamental limitations continue to influence their interpretability and translational relevance. As discussed earlier, the relatively immature phenotype of hPSC-CMs remains a major challenge, as these cells retain several fetal-like characteristics, including differences in structural organization, electrophysiological properties, and metabolic maturation compared with adult human cardiomyocytes. This immaturity complicates disease modeling, particularly for late-onset cardiac phenotypes and adult-onset electrophysiological or contractile disorders. In addition, significant protocol-dependent variability remains a critical issue, as differences in differentiation strategies, timing of signaling modulation, and culture conditions can lead to substantial heterogeneity in yield, purity, and functional output across studies. Inter-line variability further adds complexity, with genetic background-dependent differences influencing differentiation efficiency and cardiomyocyte subtype composition, even under standardized protocols. Relatedly, precise cardiac subtype specification remains incompletely controlled in many systems, limiting the ability to model chamber-specific cardiac biology and disease. Reproducibility across laboratories is also an ongoing concern, particularly given variability in reagents, extracellular matrices, and signaling factor timing. Collectively, these limitations raise important questions regarding the extent to which current in vitro hPSC-CM models faithfully recapitulate human cardiac development and disease, underscoring the need for more standardized, developmentally informed, and maturation-enhancing approaches to improve physiological fidelity and cross-study comparability.

In this context, a deeper holistic integration of developmental biology principles with stem cell biology, paired with improved standardization frameworks, is required. This includes a more comprehensive elucidation of the molecular and epigenetic programs governing cardiomyocyte maturation and subtype specification, as well as the translation of this knowledge into the development of improved differentiation protocols. Rigorous stem cell line quality control, well-standardized chemically defined protocols, optimized and tightly regulated signaling conditions, systematic benchmarking, and standardized functional validation criteria are needed to reduce variability across stem cell lines and differentiation methods. Furthermore, the use of physiologically relevant culture systems including 3D engineered heart tissues and organ-on-chip platforms will be crucial to better mimic the in vivo cardiac environment.

In conclusion, a comprehensive understanding of molecular mechanisms regulating cardiomyocyte differentiation has revolutionized the development of hPSC-based cardiac models, unlocking a wide range of promising applications. Moving forward, continued exploration of the genetic and epigenetic architecture of cardiogenesis will be pivotal in refining regenerative therapies, improving cardiac disease modeling, and advancing precision medicine approaches for cardiovascular disorders.

## Data Availability

No new data were created or analyzed in this study.

## Abbreviations

The following abbreviations are used in this manuscript:

α-MHC	α-Myosin heavy chain
β-MHC	β-Myosin heavy chain
Alk4	Activin receptor-like kinase 4
ATP	Adenosine triphosphate
AVC	Atrioventricular canal
BAF	Barrier to autointegration factor
bHLH	Basic-helix-loop-helix
BMP	Bone morphogenic proteins
Brg1	Brahma-related gene 1
CACNA	Calcium voltage-gated channel subunit alpha
cNCCs	Cardiac neural crest cells
CIBZ	CtBP-interacting BTB zinc finger protein
CM	Cardiomyocyte
CREG	Cellular Repressor of E1A-stimulated genes
CSC	Cardiac stem cells
CTGF	Connective tissue growth factor
cTnT	Cardiac troponin T
DKK-1	Dickkopf WNT signaling pathway inhibitor 1
DNMT1	DNA methyltransferase 1
DNMT3b	DNA methyltransferase 3b
Dot1L	Disruptor of telomeric silencing 1-like
E	Embryonic day
EB	Embryoid body
ELK-1	ETS transcription factor
EMT	Epithelial-to-mesenchymal transition
END-2	Endoderm-like
EOMES	Eomesodermin
ESC	Embryonic stem cell
ESC-CMs	Embryonic stem cell-derived cardiomyocytes
FGF	Fibroblast growth factors
Flk1	Fetal liver kinase 1
FOX	Forkhead box
GATA4	GATA binding protein 4
GATA6	GATA binding protein 6
GSK3	Glycogen synthase kinase 3
H3K4me3	Histone H3 tri-methylated at lysine 4
H3K27me3	Histone H3 tri-methylated at lysine 27
H3K36me3	Histone H3 tri-methylated at lysine 36
HAND2	Heart and neural crest derivatives expressed 2
Has2	Hyaluronan synthase 2
HAT	Histone acetyltransferase
HDAC	Histone deacetylase
HES1	Hairy and enhancer of split 1
hESC	Human embryonic stem cell
HEY1/2	Hairy/enhancer-of-split related with YRPW motif 1 and 2
hiPSC	Human induced pluripotent stem cell
hPSC	Human pluripotent stem cell
hPSC-CM	Human pluripotent stem cell-derived cardiomyocytes
iPSC	Induced pluripotent stem cell
iPSC-CMs	Induced pluripotent stem cell-derived cardiomyocytes
Inhbaa	Inhibin subunit beta A
IRX4	Iroquois homeobox 4
IWP-1	Inhibitor of Wnt production 1
IWR-1	Inhibitor of the Wnt response
KDR	Kinase insert domain receptor
KLF4	Krüppel-like factor 4
Jarid2	Jumonji And AT-Rich Interaction Domain Containing 2
JmjC	Jumonji C domain
lncRNA	Long non-coding RNA
LSD1	Lysine-specific demethylase 1
MEF2C	Myocyte enhancer factor 2C
MESP1	Mesoderm posterior bHLH transcription factor 1
MiRNA	MicroRNA
Mll2	Mixed-lineage leukemia 2
MLC2a	Myosin regulatory light chain 2, atrial isoform
MLC2v	Ventricular myosin light chain 2
MOZ	Monocytic leukemia zinc finger
MSX2	Msh homeobox 2
MYH6	Myosin heavy chain 6
MYH7	Myosin heavy chain 7
NFAT	Nuclear Factor of Activated T-cells
NKX2.5	NK-2 homeobox transcription factor
Nodal	Nodal growth differentiation factor
Notch	Neurogenic locus notch homolog protein
Nppa	Natriuretic peptide
NR2F2	Nuclear Receptor Subfamily 2 Group F Member 2
OCT4	Octamer-binding transcription factor 4
OFT	Outflow tract
PARP	Poly (ADP-ribose) polymerase
PBAF	Polybromo-associated BAF
PDGFRα	Platelet-derived growth factor receptor alpha
PITX2	Paired-Like homeodomain transcription factor 2
PSCs	Pluripotent stem cells
PSC-CM	Pluripotent stem cell-derived cardiomyocytes
PAX3	Paired Box 3
PBX	Pre-B-cell leukemia homeobox transcription factor
Ptip	PAX transactivation domain-interacting protein
RA	Retinoic acid
SA	Sinoatrial
SCN5A	Sodium voltage-gated channel alpha subunit 5
scRNA-seq	Single-cell RNA sequencing
SETD2	SET domain-containing protein 2
Shh	Sonic hedgehog
Sirt1	Sirtuin 1
Smad2	SMAD family member 2
SMYD1	SET and MYND domain containing 1
SOX	SRY-box transcription factor
SRF	Serum response factor
SWI/SNF	SWItch/Sucrose non-fermentable
T3	Triiodothyronine
TBX5	T-box transcription factor 5
TET2	Ten-eleven translocation 2
TnI	Troponin I
Tnnc1	Troponin C1, slow skeletal and cardiac type
Tnni3	Troponin I3, cardiac type
Tnnt2	Troponin T2, cardiac type
TWIST1	Twist family bHLH transcription factor 1
UTX	Ubiquitously Transcribed Tetratricopeptide Repeat, X Chromosome
VEGF	Vascular Endothelial Growth Factor
Whsc1	Wolf–Hirschhorn syndrome candidate 1
Wnt	Wingless-related integration site
YAP	Yes-associated protein
